# Analysis of CHK2 in vulval neoplasia

**DOI:** 10.1038/sj.bjc.6600131

**Published:** 2002-03-04

**Authors:** A Reddy, M Yuille, A Sullivan, C Repellin, A Bell, J A Tidy, D J Evans, P J Farrell, B Gusterson, M Gasco, T Crook

**Affiliations:** Ludwig Institute for Cancer Research, Imperial College Faculty of Medicine, St Mary's Campus, Norfolk Place, London W2 1PG, UK; MRC HGMP-RC, Hinxton, Cambridge CB10 1SB, UK; University Department of Pathology, Glasgow University, Western Infirmary, Glasgow, UK; Department of Gynaecological Oncology, University of Sheffield, Northern General Hospital, Sheffield S5 7AU, UK; Department of Histopathology, St Mary's Hospital Medical School, Norfolk Place, London W2, UK; UO Oncologia Medica, Azienda Ospedaliera S. Croce e Carle, Via Coppino 26, 12100, Cuneo, Italy

**Keywords:** vulval cancer, p53, CHK2

## Abstract

Structure and expression of the Rad53 homologue CHK2 were studied in vulval neoplasia. We identified the previously described silent polymorphism at codon 84 (A>G at nucleotide 252) in the germ-line of six out of 72, and somatic mutations in two out of 40 cases of vulval squamous cell carcinomas and none of 32 cases of vulval intraepithelial neoplasia. One mutation introduced a premature stop codon in the kinase domain of CHK2, whereas the second resulted in an amino acid substitution in the kinase domain. The two squamous cell carcinomas with mutations in CHK2 also expressed mutant p53. A CpG island was identified close to the putative CHK2 transcriptional start site, but methylation-specific PCR did not detect methylation in any of 40 vulval squamous cell carcinomas, irrespective of human papillomavirus or p53 status. Consistent with this observation, no cancer exhibited loss of CHK2 expression at mRNA or protein level. Taken together, these observations reveal that genetic but not epigenetic changes in CHK2 occur in a small proportion of vulval squamous cell carcinomas.

*British Journal of Cancer* (2002) **86**, 756–760. DOI: 10.1038/sj/bjc/6600131 www.bjcancer.com

© 2002 Cancer Research UK

## 

Although vulval squamous cell carcinoma (SCC) is less common than cervical cancer, it is nevertheless of interest since a proportion of cases contain sequences from high-risk human papillomavirus (HPV) types (principally HPV 16), whereas a further, substantial subset of cancers arise via HPV-independent pathways. Pathobiological differences exist between HPV positive and HPV negative cancers (Crum, 1992), but allelotype analysis suggests that there are no significant differences in sites of loss of heterozygosity (LOH) (Pinto *et al*, 1999). A number of studies have however revealed that mutation in p53 is more common in HPV negative cancers (Lee *et al*, 1994; Brooks *et al*, 2000). Mechanistically, it is hypothesised that mutation in p53 functionally compensates for the absence of HPV 16E6, since this protein mediates inactivation of p53 via promotion of ubiquitin-dependent proteolysis. Despite the more common mutation of p53 in HPV negative cases, a substantial number of vulval SCC occur which lack both mutation and HPV. The mechanism, if any, by which p53 function is compromised in such cases is not known.

The CHK2 kinase, the human homologue of yeast RAD53, is located on chromosome 22q which is a common site for LOH in vulval cancer (Worsham *et al*, 1991; Pinto *et al*, 1999). CHK2 functions downstream of ATM (ataxia telangiectasia-mutated protein) in response to DNA damage to phosphorylate p53 and BRCA1 and thereby regulate the tumour suppressor functions of these proteins (Chehab *et al*, 2000; Lee *et al*, 2000; Matsuoka *et al*, 2000). Furthermore, CHK2-deficient mice fail to maintain G2 arrest following irradiation (Hirao *et al*, 2000). Taken together these data suggest that CHK2 functions at both G1 and G2 cell cycle checkpoints. The protein contains functionally important fork head-associated and kinase domains.

Somatic mutations in CHK2 have been reported in both solid tumours (Haruki *et al*, 2000) and in myelodysplastic syndrome (Hofmann *et al*, 2001). Furthermore, analysis of individuals with the Li–Fraumeni cancer predisposition syndrome revealed that a subset of individuals with the syndrome, but lacking mutations in p53, harbour heterozygous germ-line mutations in CHK2 (Bell *et al*, 1999). These observations support the assertion that p53 and CHK2 function in a common pathway of tumour suppression and raise the interesting possibility that abrogation of CHK2 function via mutation or loss of expression might functionally compensate for mutations in cancers with wild-type p53. To investigate this hypothesis, we have performed analysis of the structure and expression of CHK2 in a series of vulval cancers and pre-malignant lesions characterised for both HPV and p53 status.

## MATERIALS AND METHODS

### Tissues

Fresh-frozen tumour tissue was available from 40 cases of vulval SCC, each with matched normal vulval epithelium. The diagnosis in each case was determined by histopathological analysis. Genomic DNA was isolated from frozen tissues (normal and tumour) by proteinase K digestion and RNA by RNAzol B. Paraffin sections of vulval intraepithelial neoplasia (VIN) were obtained from the pathology archives of St Mary's Hospital (London). The diagnosis and presence of adequate neoplastic tissue was verified for each case and DNA isolated from suitable samples by extended digestion in proteinase K.

### Gene analysis

Each genomic DNA was initially checked by amplification of globin using the PCO3/PCO4 primer pair (Greer *et al*, 1991). HPV DNA was sought using the HPV consensus primer pair CPI/CPIIG which detects a broad range of HPV types and allows detection of HPV in DNA from paraffin sections (Smits *et al*, 1992). Positive cases were typed by direct sequencing of amplified products. The p53 status of each cancer was determined by SCCP and DNA sequencing as described previously (Brooks *et al*, 2000). Mutations in CHK2 were sought in genomic DNA by SSCP and, in cases where RNA was available, by RT–PCR SSCP. In total, 36 SCC were examined by RT–PCR SSCP. Primers for SSCP from genomic DNA were designed from AL117330 and AL121825 (Table 1

**Table 1:**
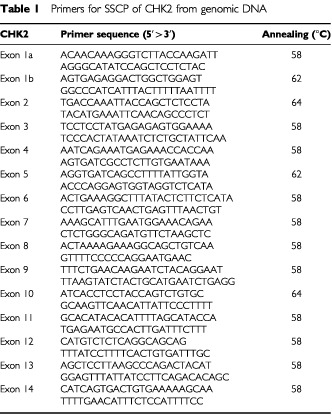
Primers for SSCP of CHK2 from genomic DNA

). Primers for RT–PCR SSCP were as described (Haruki *et al*, 2000). cDNA was synthesized in 50 μl reaction from 3 μg of total RNA using the Stratagene ProStar system. Two μl of this solution was used for each PCR. In all cases, PCR products were resolved on 6% native polyacrylamide gels with and without 10% glycerol. When SSCP amplimers exhibited aberrant mobility, the fragment was re-amplified with *Pfx* DNA polymerase and multiple plasmid clones in the pCRBlunt vector were sequenced.

### Analysis of CpG methylation in CHK2

A CpG island is located in AL117330 between 26038–26731. Using a combination of 5′RACE and searching of the human genome database (http://genome.ucsc.edu/goldenPath/hgTracks.html), we established that this is close to the 5′ end of the CHK2 mRNA. Methylation in the CHK2 promoter was analysed using MSP (Herman *et al*, 1996). Primers were designed from the sequence of the CpG island using a commercially available software programme (Intergen). Primer sequences are as follows: Unmethylated 5′-TACAACAACCCATAAAACCCCAAACAAA-3′ and 5′-TAGATTTTGATGTGTTTTTTGTTTGGGTTT-3′, giving a product of 161 bp. Methylated 5′-GACGACCCATAAAACCCCGAACGAA-3′ and 5′-TTTCGACGTGTTTTTCGTTCGGGTTC-3′, giving a product of 154 bp. Following PCR, reactions were resolved on 2% agarose gels and visualised under UV. Each PCR included control methylated and unmethylated DNA samples.

### Immunocytochemistry

Five-μm sections were cut from archival paraffin blocks, stained with haematoxylin and eosin, and the diagnosis in each case verified by two pathologists. For immunocytochemical analysis, the sections were subjected to antigen retrieval by microwaving in citrate buffer, then incubated with anti-human CHK2 antibody (Santa Cruz, sc-8812). The specificity of this antibody was confirmed by Western blotting prior to immunocytochemical studies. Sections were reviewed by two pathologists to score expression. A negative control section, lacking the primary antibody, was included in each immunocytochemical analysis.

## RESULTS

### Somatic mutations in CHK2 in vulval neoplasia

**Figure 1:**
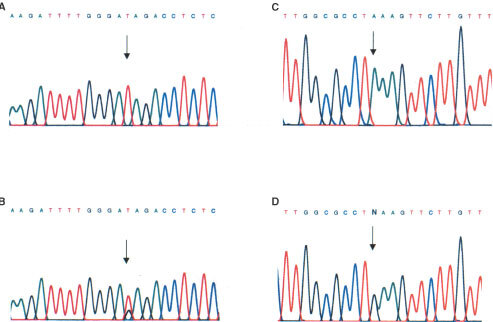
Somatic mutation in CHK2 in vulval cancer. (**A**) Sequence analysis of plasmid clone containing the arrowed mutation at codon 377 of CHK2 GAG>TAG (Glu>Ter); (**B**) Direct sequencing of cDNA from this tumour reveals loss of heterozygosity; (**C**) Sequence of plasmid clone containing the arrowed mutation at codon 394 of CHK2 GAA>AAA (Glu>Lys); (**D**) Direct sequencing of cDNA from this tumour reveals no evidence for loss of heterozygosity.

We performed analysis of the sequence of CHK2 in vulval neoplasia. Primers for analysis of CHK2 sequence by SSCP from genomic DNA were designed by reference to the intron–exon structure of the gene (Accession No. AL117330, and AL121825). Primer pairs and annealing temperatures are shown in Table 1. The previously described silent polymorphism at nucleotide 252 in codon 84 (GAA>GAG) (Bell *et al*, 1999; Haruki *et al*, 2000; Hofmann *et al*, 2001) was observed in four out of 40 individuals with vulval SCC and two of 32 cases of VIN. Analysis of matched normal epithelium from each individual with SCC revealed that each was heterozygous for this polymorphism in the germline. Mutations in CHK2 were detected in two vulval SCC by RT–PCR SSCP and subsequent sequencing of cloned RT–PCR products (Figure 1

and Table 2

**Table 2:**
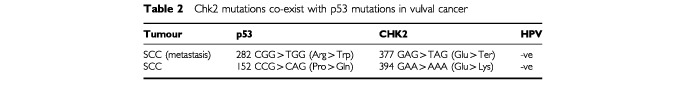
Chk2 mutations co-exist with p53 mutations in vulval cancer

). Both mutations occurred in the kinase domain of CHK2. One introduced a premature stop at codon 377 (gAg>TAg), whereas the second was a substitution (gAA>AAA; Glu>Lys) at codon 394 (Table 2). During these studies a number of highly conserved 3′ fragments of CHK2 were identified which map to several autosomes and the X chromosome (Sodha *et al*, 2000). These fragments are homologous to sequences in exons 10–14 of CHK2. We therefore performed additional analyses to confirm that the sequence changes we had detected represent *bona fide* CHK2 mutations rather than rare polymorphisms within the homologous fragments. We used forward primers located upstream of exon 9 which are unique to CHK2 together with reverse primers from exons 10 and 11 to amplify CHK2-specific genomic sequences and sequenced these. These confirmed the presence of the mutations. We also performed sequence analysis of matched normal tissue for each cancer with mutation. The mutations were not detected in the normal tissue, confirming their authenticity as somatic CHK2 mutations. We were interested to determine whether there was loss of heterozygosity (LOH) in the cases with CHK2 mutations. In an attempt to address this, we performed direct sequencing of the RT–PCR products in the two cases with mutations. In one case (codon 377 gAg>TAg) the mutated allele was clearly the predominant one present, consistent with loss of the wild-type allele (Figure 1B). In the remaining case, there was no evidence of LOH, both alleles being equally represented (Figure 1D). It should be noted, however, that the presence of normal tissue may mask LOH. As such, we cannot be certain that this is a heterozygous mutation.

### CHK2 mutations co-exist with p53 mutations in vulval SCC

The first reported examples of human–tumour associated CHK2 mutations were in Li–Fraumeni patients in which p53 was wild-type (Bell *et al*, 1999). We therefore checked the p53 sequence in the vulval SCC with CHK2 mutations. Both cancers with mutations in CHK2 were mutant for p53 (Table 2).

### The CHK2 promoter is not hypermethylated in vulval neoplasia

**Figure 2:**
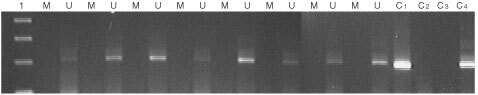
Absence of CpG methylation in the CHK2 promoter in vulval cancer. MSP was performed on bisulphite-treated genomic DNA isolated from vulval SCC, using the primers for MSP as described in Materials and Methods. Lane 1 is 100 bp ladder. For each carcinoma M=methylated, U =unmethylated. C_1_= unmethylated control DNA with U primers; C_2_=unmethylated control DNA with M primers; C_3_=methylated control DNA with U primers; C_4_=methylated control DNA with M primers.

We were interested to determine whether CHK2 might be inactivated by alternative mechanisms in this series of cancers. Using a combination of data base searching and 5′ RACE, we mapped the 5′ end of the CHK2 mRNA and the CHK2 promoter. These studies identified a CpG island 5′ of, and including the non-coding first exon of the CHK2 mRNA. We designed primers for MSP to determine whether aberrant hypermethylation occurred in the CpG island in vulval cancer. The precise positions of the primers are given in Materials and Methods. We did not detect methylation in the CHK2 promoter in any of the 40 vulval SCC analysed, despite detection of methylation in the control DNA samples (Figure 2

).

### CHK2 expression is not down regulated in vulval neoplasia

**Figure 3:**
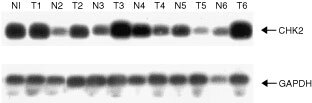
RT–PCR analysis of CHK2 expression in vulval neoplasia. RT–PCR was performed on RNA isolated from matched pairs of normal vulval epithelium (N) and tumour (T). CHK2 and the control RNA (GAPDH) are indicated.

**Figure 4:**
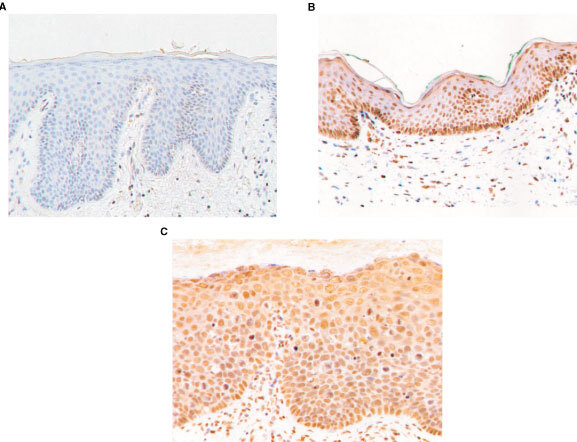
Immunocytochemical analysis of expression of CHK2 in vulval neoplasia. The sections shown are (**A**) control with no primary antibody; (**B**) normal vulval epithelium; (**C**) VIN II. Sections were prepared and stained as described in Materials and Methods.

Despite the absence of methylation in the CHK2 promoter, it was clearly important to examine expression of the gene in cancers. We therefore performed both RT–PCR and immunocytochemical analyses to compare expression in normal and neoplastic vulval epithelium. Expression levels of CHK2 assessed by RT–PCR were variable between normal and tumour pairs (Figure 3

). However, we did not identify a single case in which CHK2 expression was lost. To further confirm that loss of expression does not occur commonly in vulval cancer, we performed immunocytochemical analysis of a series of vulval neoplastic lesions. These samples comprised six cases of vulval SCC, four cases of VIN I, six cases of VIN II and 22 cases of VIN III. There was expression of CHK2 protein throughout normal vulval epithelium (Figure 4B

) but no loss of expression in any of the VIN or vulval SCC analysed (Figure 4C).

### Discussion

Abrogation of p53 function is a critical event in tumorigenesis. p53 is directly phosphorylated by CHK2 in response to DNA damage. Furthermore, germ-line mutations in CHK2 occur in individuals with Li–Fraumeni syndrome which express wild-type p53. Taken together, these observations suggested that loss of function in CHK2 might represent an alternative mechanism by which the p53 pathway can be inactivated or attenuated in cancers lacking other recognised means of abrogation of p53 function. The mechanism by which p53 function is compromised in vulval cancer is of considerable interest since only a subset of cancers have HPV or p53 mutation (Crum, 1992; Lee *et al*, 1994; Brooks *et al*, 2000).

We show in this report that somatic mutation of CHK2 occurs in a small proportion of vulval cancers. Both of the mutations we detected are in the kinase domain of CHK2, and, therefore highly likely to compromise the function of the protein. One of the mutants introduced a premature termination codon whereas the other mutation resulted in an amino acid substitution. Interestingly, both cancers with CHK2 mutations also contained mutant p53. The simultaneous presence of mutations in CHK2 and in p53 has been reported previously in colon cancer (Bell *et al*, 1999) and small cell lung cancer (Haruki *et al*, 2000) but not in a case of myelodysplastic syndrome with CHK2 mutation in which p53 was wild-type (Hofmann *et al*, 2001). Taken together with the present analysis of vulval cancer, these data imply that CHK2 and p53 mutations are not mutually exclusive events in cancer. Our results also suggest that mutation of CHK2 is not a common mechanism by which vulval cancers negative for HPV and lacking p53 mutations abrogate the tumour suppressor function of p53.

To investigate alternative potential mechanisms by which CHK2 might be altered in vulval neoplasia, we analysed the CHK2 promoter and 5′ end of the CHK2 transcript. The CpG island located close to the transcription start site of CHK2 represents a potential site for aberrant methylation and transcriptional silencing, but we found no evidence for CpG hypermethylation in any of the vulval SCC in our series. Consistent with this, there was abundant expression of CHK2 at both mRNA and protein levels in normal, pre-malignant and malignant vulval epithelium and no case was observed in which expression was lost.

In conclusion, our results reveal that CHK2 is a target for somatic mutation in a small proportion of cases of vulval cancer and further confirm its status as a *bona fide* human tumour suppressor gene. They do not, however, support a model in which mutation or loss of expression of CHK2 might functionally substitute for inactivation of p53 via mutation or expression of HPV E6.
